# Catalytic activity of Co-nanocrystal-doped tungsten carbide arising from an internal magnetic field†

**DOI:** 10.1039/d1ra01181b

**Published:** 2021-04-14

**Authors:** M. Morishita, A. Nozaki, H. Yamamoto, N. Fukumuro, M. Mori, K. Araki, F. Sakamoto, A. Nakamura, H. Yanagita

**Affiliations:** Department of Chemical Engineering and Materials Science, University of Hyogo 2167 Shosha Himeji 671-2280 Japan morisita@eng.u-hyogo.ac.jp; Graduate Student of University of Hyogo 2167 Shosha Himeji 671-2280 Japan; Sanalloy Industry Co., Ltd 290-44 Takahashi, Fukusaki-cho Kanzaki 679-2216 Japan

## Abstract

Pt is an excellent and widely used hydrogen evolution reaction (HER) catalyst. However, it is a rare and expensive metal, and alternative catalysts are being sought to facilitate the hydrogen economy. As tungsten carbide (WC) has a Pt-like occupied density of states, it is expected to exhibit catalytic activity. However, unlike Pt, excellent catalytic activity has not yet been observed for mono WC. One of the intrinsic differences between WC and Pt is in their magnetic properties; WC is non-magnetic, whereas Pt exhibits high magnetic susceptibility. In this study, the WC lattice was doped with ferromagnetic Co nanocrystals to introduce an ordered-spin atomic configuration. The catalytic activity of the Co-doped WC was ∼30% higher than that of Pt nanoparticles for the HER during the hydrolysis of ammonia borane (NH_3_BH_3_), which is currently attracting attention as a hydrogen fuel source. Measurements of the magnetisation, enthalpy of adsorption, and activation energy indicated that the synergistic effect of the WC matrix promoting hydrolytic cleavage of NH_3_BH_3_ and the ferromagnetic Co crystals interacting with the nucleus spin of the protons was responsible for the enhanced catalytic activity. This study presents a new catalyst design strategy based on the concept of an internal magnetic field. The WC–Co material presented here is expected to have a wide range of applications as an HER catalyst.

## Abstract 2

The catalytic activity of the Co-doped WC is 30% higher than that of Pt nanoparticles for the hydrogen evolution reaction arising from an internal magnetic field.

## Graphical abstract

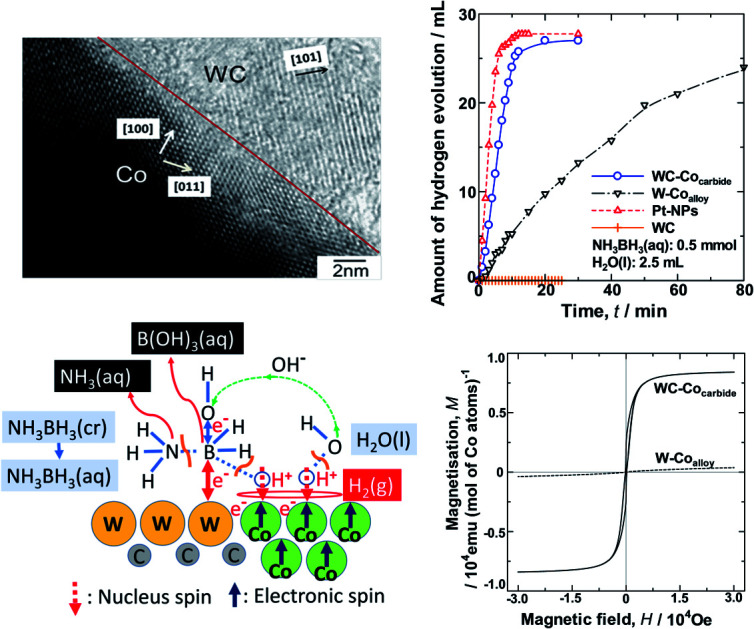

## Introduction

The development of catalysts for the hydrogen evolution reaction (HER) is critical for producing hydrogen fuel as a substitute to fossil fuels to reduce global CO_2_ emissions. Tungsten carbide (WC) has 10 valence electrons, 5d^6^ from W and 2p^4^ from C, similar to Pt (5d^10^). Since 1973, when Levy and Boudart^1^ proposed that WC exhibited singular catalytic activity similar to Pt, this topic has been investigated by many researchers. Bennett *et al.*^2^ measured the valence band spectrum of WC using X-ray photoelectron spectroscopy (XPS) and compared it with the spectra of W and Pt. Near the Fermi level (*E*_F_), the electronic density of states (DOS) of WC more closely resembled that of Pt^3^ than W.^4^ Houston^5^ compared the DOS of W, WC, and Pt using soft X-ray appearance potential spectroscopy (SXAPA). Contrary to the results of previous studies,^3,4^ the width of the unoccupied portion of the 5d band of W in WC was larger than in pure W.^5^ Another study^6^ showed that the differences between the DOS measured by SXAPA^5^ and predicted using a rigid band model^1,2^ resulted in different crystal structures of body centred cubic (bcc) W, face centred cubic (fcc) Pt, and hexagonal WC. Mattheiss and Hamann^7^ investigated the DOS of the bulk and (0001) surface of WC using a relativistic linear augmented-plane wave method.^8^ The DOS near the *E*_F_ of the (0001) surface associated with the catalytic properties was larger than in the bulk. Recently, the electronic states of Pt were updated^9^ using the projector-augmented-plane wave method implemented in the VASP code^8^ using the generalised gradient approximation (Perdew–Burke–Ernzerhof version) (GGA-PBE),^10^ which clarified that *E*_F_ is located at the top of the 5d DOS, consistent with its highest catalytic activity of all elements.

Esposite *et al.*^11^ showed that a monolayer film of Pt deposited on a bulk WC substrate exhibited a similar electrochemical hydrogen overpotential, *η*_H_2__, to that of Pt in an aqueous H_2_SO_4_ solution, indicating that the surface electronic and chemical properties of monolayer Pt on a bulk WC substrate are significantly similar to those of bulk Pt. Zhang *et al.*^12^ synthesised a Fe_3_C/Co_3_C/WC/C carbide composite prepared by combining hydrothermal synthesis with resin impregnation and pyrolysis. Linear-sweep voltammetry of the Fe_3_C/Co_3_C/WC/C in an aqueous KOH solution exhibited similar behaviour to that of a Pt/C electrode, indicating that it is a candidate cathode material for polymer electrolyte membrane fuel cells (PEMFCs).^13^ In addition, a 7.5 wt% Pt/W_2_C catalyst showed a current density 2–3 times higher than that of a commercial 20 wt% Pt/C for electrolysis in H_2_SO_4_ aqueous solution due to the synergistic effect of Pt and WC.^13^ Good catalytic activity for the hydrogen evolution reaction (HER) *via* electrolysis of the H_2_SO_4_ aqueous solution was observed using a powder microelectrode composed of 16.20 wt% Pt/WC^14^ or a bimetallic carbide composed of Mo_2_C and WC.^15^ Zheng *et al.* demonstrated that PEMFCs using a Nafion membrane containing WC nanoparticles exhibited enhanced power density and durability over 100 h of use.^16^

The catalytic activities of WC and its composites have only been observed under conditions of an applied external voltage,^11–16^ and the predicted intrinsic catalytic activity of mono WC under voltage-free operation has not yet been verified. Although Levy and Boudart observed that WC catalysed the reduction of WO_3_ with hydrogen gas in the presence of water, and the isomerisation of 2,2-dimethylpropane to 2-methybutane, the rates were merely 0.37% and 0.01%, respectively, in comparison with the performance of Pt.^1^

Bennett *et al.*^2^ noted that one of the intrinsic differences between WC and Pt is their magnetic properties; WC is non-magnetic, whereas Pt exhibits high magnetic susceptibility. Cerri *et al.*^17^ studied the magnetic properties of polycrystalline Gd and found that hydrogen chemisorption induced a disordering of the electronic spin polarisation on the surface. An FTIR spectroscopy study confirmed that antiferromagnetic LaFeO_3_ accelerated the nucleus spin conversion of ortho liquid hydrogen (composed of two antiparallel nucleus spins) to para liquid hydrogen (composed of two parallel nucleus spins) to reach equilibrium.^18^ Furthermore, Galces-Pineda *et al.*^19^ observed that an external magnetic field accelerated oxygen evolution during the electrolysis of water.

An application of HER catalysts is the generation of hydrogen fuel from NH_3_BH_3._ In its stable crystal form, NH_3_BH_3_ contains 19.6 wt% hydrogen,^20^ and is being investigated for efficient transportation of hydrogen-based fuel for portable fuel-cell systems. Previous studies investigated the HER by hydrolysis over 10 wt% Co^21^ or 2 wt% Pt^22^ (both supported by Al_2_O_3_) and found that the HER in the latter was significantly faster than that in the former. A similar HER in NH_3_BH_3_(aq) catalysed by Ni nanoparticles (NPs) supported by a zeolite molecular sieve was observed.^23^

In this study, the WC lattice was doped with ferromagnetic Co nanocrystals (WC–Co_carbide_)^24^ to introduce an ordered-spin configuration as an internal magnetic field to avoid the need for an external applied voltage. The catalytic performance of WC–Co_carbide_ for the HER in NH_3_BH_3_(aq) was compared with that of Pt nanoparticles (PT_NPs), commercial mono WC, and a bcc W–Co solid solution (W–Co_alloy_) in the spin glass state. The present study aims to investigate the (1) effect of an internal magnetic field instead of an external voltage on the catalytic activity; (2) kinetics of the HER; and (3) practical applications of the catalyst.

## Materials and methods

The W–Co_alloy_ powder consisting of a solid solution of Co-supersaturated bcc W was prepared using a hydrothermal synthesis method that we have described previously.^24^ The precursor materials were 99% ammonium tungstate pentahydrate (5(NH_4_)_2_O·12WO_3_·5H_2_O; Kanto Chemical Co. Inc., Japan) and 99% cobalt acetate tetrahydrate (Co(C_2_H_3_O_2_)_2_·4H_2_O; Kojundo Chemical Laboratory Co. Ltd, Japan), which were mixed to achieve a molar ratio of W : Co = 80 : 20. Briefly, W–Co_alloy_ was carburised at 1173 K with a gas with a composition of 23 vol% CO_2_, 32 vol% H_2_, and 45 vol% Ar to form WC containing Co nanocrystals (WC–Co_carbide_). In this study, CO_2_ was used as the carburisation gas instead of CO to increase the safety of the process. Since CO_2_ barely carburises the W–Co_alloy_ based on the thermochemical equation, CO_2_ was converted into CO *in situ* in the furnace using 50 : 50 mol% Fe–Al powder, where the Al thermally reduces CO_2_ into CO. Although 10.8 ks was shown to be a sufficient processing time for carburisation using CO,^24^ a total of 32.4 ks was required when CO_2_ was used. During this carburisation procedure, the samples were ground at each 10.8 ks interval.

The chemical composition and structure of WC–Co_carbide_ were confirmed by X-ray diffraction (XRD; Rigaku, Ultima IV) and electron probe microanalysis (EPMA; JOEL, JXA-8900) operated at a 15 kV accelerating voltage. The nanostructure of the Co crystal was observed by high-resolution transmission microscopy (HRTEM; JOEL, JEM-2100) operated at an accelerating voltage of 200 kV. The metallic state of the surface of the Co crystal was confirmed *via* XPS (ULVAC-PHI Inc., PHI5000) using monochromatic X-rays (Al Kα, 1486.6 eV). The specific surface area (SSA) of all samples was measured using the Brunauer–Emmett–Teller method (Shimadzu, TriStar II 3020) with the Kr physisorption isotherm obtained at 77 K.

A standard catalyst containing 1 wt% Pt_NPs on a carrier of Al_2_O_3_ particles (Catalysis Society of Japan)^25^ was used to obtain comparative data for the catalytic reaction. The SSA of the Pt was 3.40 m^2^ g_cat_^−1^ and the total SSA of the catalyst, including the Al_2_O_3_, was 176 m^2^ g_cat_^−1^.^25^ A commercial mono WC particle (Kojundo Chemical Laboratory Co. Ltd, Japan; 99%) was also used as a reference sample.

The WC–Co_carbide_, W–Co_alloy_, Pt_NPs, and WC samples were flushed in a H_2_ atmosphere at 473 K for 2 h. Then, 20 mg of each sample was placed in a glass test tube with 1 ml of H_2_O. In addition, 0.5 mmol of NH_3_BH_3_(cr) was dissolved in 1.5 ml of H_2_O to form an aqueous solution of NH_3_BH_3_(aq) that was mixed with each of the aforementioned samples to evolve hydrogen by hydrolysis of the solution. Four repeat measurements of the hydrogen evolution volume (HEV) were performed for the WC–Co_carbide_, W–Co_alloy,_ and Pt_NPs samples, whereas only one measurement was considered sufficient for WC as it showed no catalytic activity.

The HER rate for each sample was evaluated by determining the slope of the HEV curve as a function of time using the least squares method. The average HER rates of the four repeat measurements are presented here. The 1-sigma error (68% confidence interval)^26^ was calculated by dividing the standard deviation (*σ*) of the four measurements by the average value.

To determine *E*_a_, the HEV over WC–Co_carbide_ was measured at 308, 318, 328, and 338 K using a solution prepared with 1.0 mmol NH_3_BH_3_(cr) and 1.5 ml of H_2_O. A higher solution concentration than that used in the previous measurements was selected to achieve a longer reaction time and hence, more accurate Arrhenius plots. Three HER measurements were performed at each temperature and the average HER rate is presented. The 1-sigma error was evaluated by dividing 1*σ* of the three measurements by the average value at each temperature. The 1-sigma error was evaluated from the standard deviation of the 12 total measurements.

To determine the value of *M* of WC–Co_carbide_, the magnetic hysteresis loop was measured using a SQUID instrument (QD, MPMS) under a magnetic field increased to a maximum of 3 × 10^4^ Oe. Temperatures of 308 and 4 K were used to investigate the maximum value of *M*,^27,28^ respectively.

The standard enthalpies of adsorption of H_2_(g), Δ_ad_*H*^°^_m_, for WC–Co_carbide_, WC, and a commercial 99.9% Pt powder (Kojundo Chemical Laboratory Co. Ltd, Saitama) were measured at 423 K using a Calvet-type microcalorimetre (SETRAM, C80) in a H_2_ atmosphere.

## Results and discussion

### Hydrogen evolution over WC–Co

**Fig. 1:**
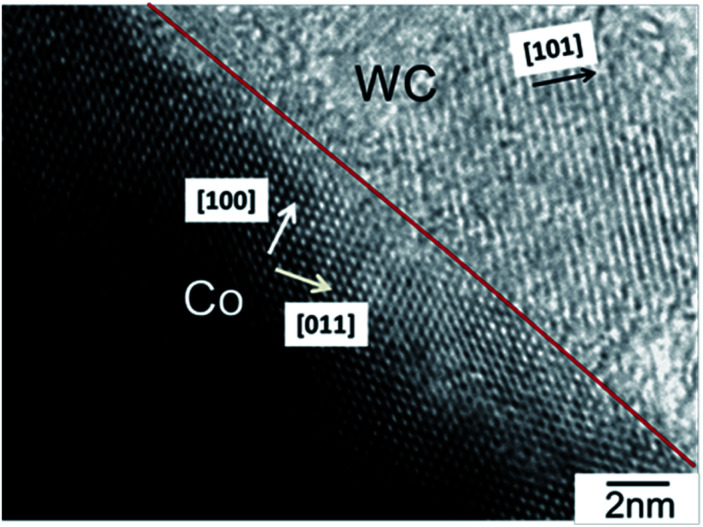
Bright-field TEM image of WC–Co_carbide_.

Fig. 1 shows a representative bright-field TEM image of a WC–Co_carbide_ sample. Co crystals with the fcc structure, [100] orientation, and diameter of 60 nm were observed in the WC matrix with [101] orientation. The atomic configuration was the same as that observed in our previous study.^24^ The SSA values of the WC–Co_carbide_, W–Co_alloy_, and Pt_NPs samples are shown in Table 1. The WC–Co_carbide_ exhibited an SSA ∼ 60% smaller than that of the Pt_NPs, whereas WC–Co_carbide_ exhibited a smaller SSA than that of W–Co_alloy_.

**Table 1:** Summary of the SSA and HER results extracted from Fig. 2 and 3 for all sample types

Sample	SSA (m^2^ g_cat_^−1^)	*N*_HER_ (H_2_ mol min^−1^ m^−2^)	*N* (NH_3_BH_3_(aq) mmol^−1^)	*V* (H_2_O mL^−1^)	*T* (K)
WC–Co_carbide_	1.35	3.76 ± 0.37	0.5	2.5	308
WC–Co_carbide_	1.35	7.63 ± 0.32	1.0	2.5	308
WC–Co_carbide_	1.35	12.14 ± 2.42	1.0	2.5	318
WC–Co_carbide_	1.35	26.92 ± 3.13	1.0	2.5	328
WC–Co_carbide_	1.35	46.91 ± 7.37	1.0	2.5	338
W–Co_alloy_	2.23	0.45 ± 0.11	0.5	2.5	308
Pt_NPs	3.40	2.90 ± 1.04	0.5	2.5	308
Comm. WC	1.03	0	0.5	2.5	308
Comm. Pt	0.12	—	—	—	—

Fig. S1 (see ESI†) shows the XRD results. Only peaks related to the WC matrix and Co crystals were observed. Fig. S2 (see ESI†) shows the Co 2p XPS spectrum of WC–Co_carbide_. The peak at 778.4 eV was assigned to the metallic state Co^0^. Fig. S3 (see ESI†) shows representative EPMA/SEM images of W Lα, C Kα, and Co Kα for the WC–Co_carbide_ particle. Because W, C, and Co were distributed homogeneously, HRTEM observation (Fig. 1) was necessary to distinguish the Co crystals from the WC matrix.

**Fig. 2:**
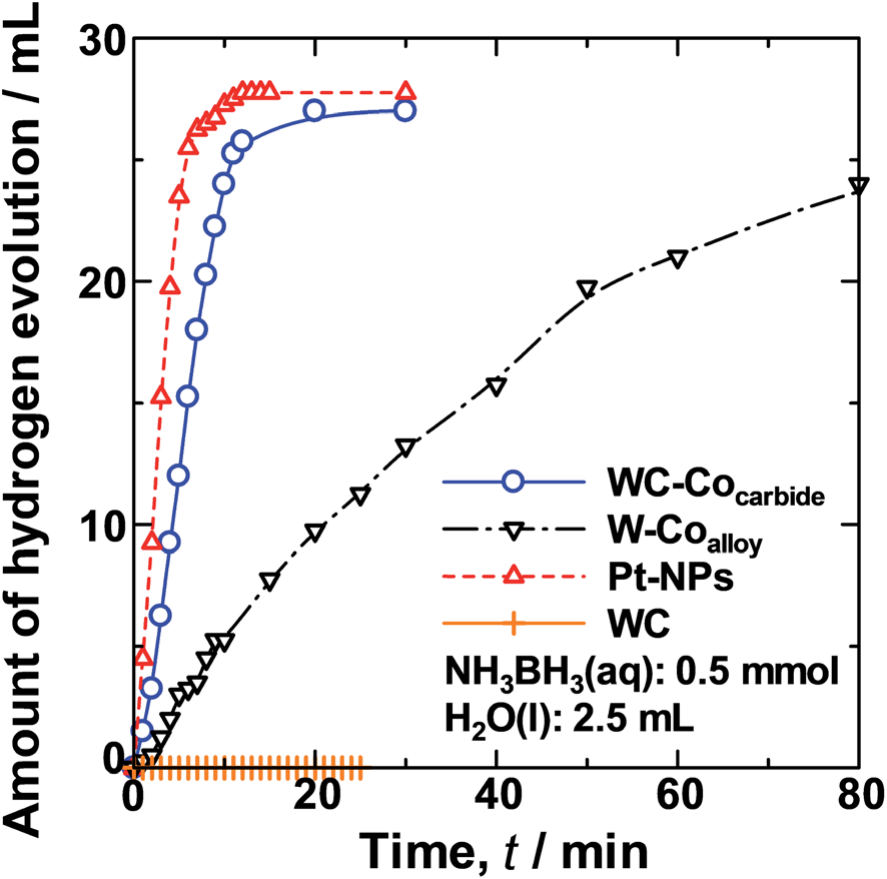
Comparison of the HEV over time from hydrolysis of a NH_3_BH_3_(aq) solution (0.5 mmol, 2.5 ml) at 308 K using the various catalyst samples.

Fig. 2 shows the change in the average HEV over time (*t*) for hydrolysis of the NH_3_BH_3_(aq) over WC–Co_carbide_, W–Co_alloy_, Pt_NPs, and commercial WC samples. The slope of the HEV(*t*) curve for WC–Co_carbide_ was steeper than that for Pt_NPs, indicating that WC–Co_carbide_ exhibited singular catalytic activity, whereas W–Co_alloy_ exhibited less activity.

The normalised HER rates per unit area, *N*_HER_ (H_2_ mol min^−1^ m^−2^), determined by normalising the HEV by the SSA values are shown in Table 1, along with their error values. The *N*_HER_ of WC–Co_carbide_ was ∼30% higher than that of Pt_NPs, indicating that it exhibits singular catalytic activity similar to Pt. The *N*_HER_ of W–Co_alloy_ was only approximately 10% of that of WC–Co_carbide_.

### Activation energy

**Fig. 3:**
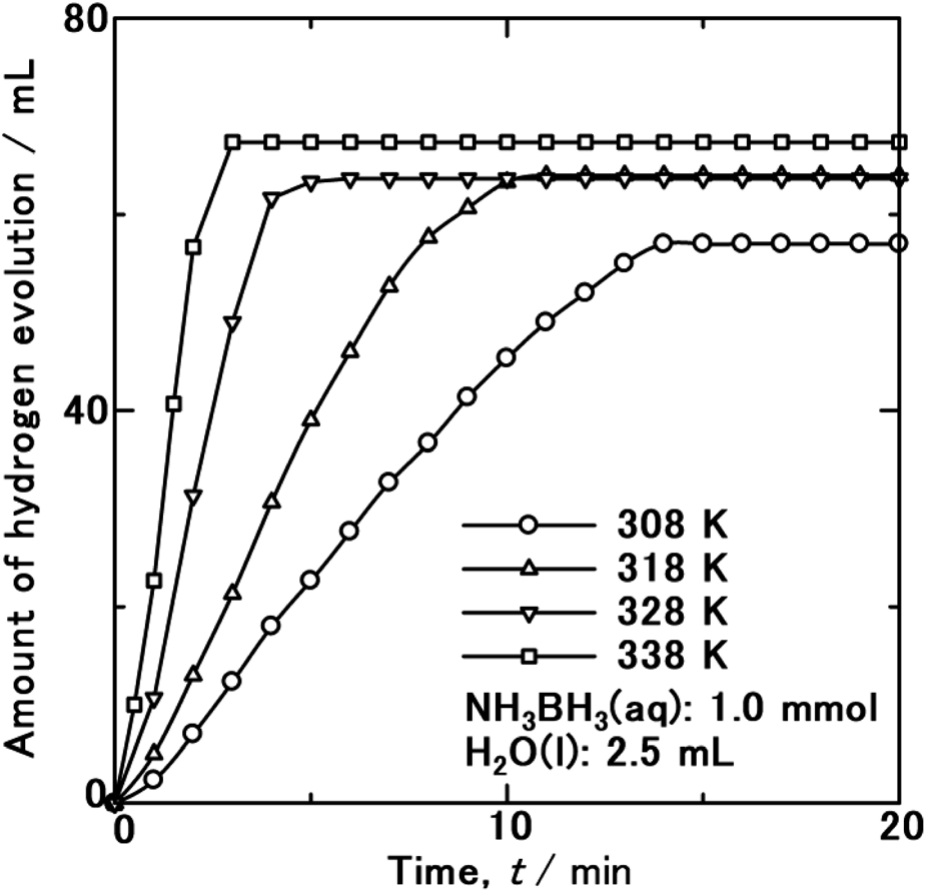
HEV from hydrolysis of NH_3_BH_3_ aqueous solution (1 mmol, 2.5 ml) using WC–Co_carbide_ as a catalyst at various temperatures.

To clarify the mechanism of the singular catalytic activity of WC–Co_carbide_, its *E*_a_ value was investigated. Fig. 3 shows the HEV as a function of time during the hydrolysis of NH_3_BH_3_(aq) over WC–Co_carbide_ at 308, 318, 328, and 338 K. Comparing the data measured at 308 K to the corresponding curve in Fig. 2, the HEV at the same time was consistent with doubling the NH_3_BH_3_ concentration. Hence, a large number of reaction sites were available for studying the HER. The slopes of the HEV(*t*) curves increased with increasing temperature, indicating that the hydrolysis was a result of a thermally activated process.

**Fig. 4:**
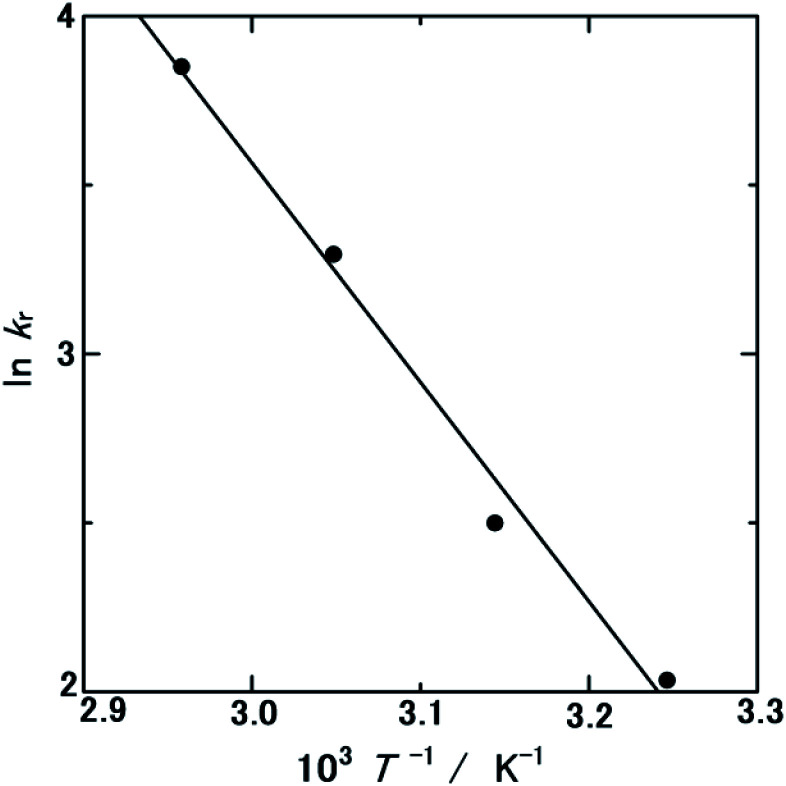
ln *k*_r_*versus T*^−1^ plot calculated from Table S1.†

The *E*_a_ is defined by the Arrhenius equation:1
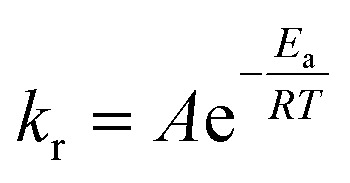
where *k*_r_ is the reaction rate constant, *R* is the gas constant (8.3145 J K^−1^ mol^−1^), and *A* is the frequency factor indicating the number of collisions of the reaction species. The *N*_HER_ values summarised in Table 1 were regarded as *k*_r_ which were plotted as an Arrhenius plot in Fig. 4 (ln *k*_r_*vs. T*^−1^). The ln *k*_r_ values decreased linearly as a function of *T*^−1^, where *E*_a_ was calculated from the slope of this curve. The *E*_a_ and *A* values for the HER over WC–Co_carbide_ were 54.0 ± 13.0 kJ mol^−1^ and 1.01 × 10^10^ ± 2.44 × 10^9^ mol m^−2^ min^−1^, respectively, where the uncertainty was evaluated as the error propagation^26^ of the uncertainties of the *N*_HER_ data at each temperature listed in Table 1. This *E*_a_ value was larger than the literature value for Pt NPs (2 wt% Pt on an Al_2_O_3_ support)^22^ and similar to that of Co NPs (10 wt% Co on an Al_2_O_3_ support),^21^ indicating that the hydrogen was released from the surface of the Co nanocrystals in the WC matrix.

The catalytic activity of WC–Co_carbide_ was evaluated considering the hydrogen overpotential (*η*_H2_). The *η*_H2_of Pt is 0 V by definition, whereas for Co it is in the range of −0.25 to −0.47 V in 1 M H_2_SO_4_ aqueous solution at 303 K under a current density of 0.3–10 mA cm^−2^.^29^ Chandra and Xu^22^ determined *E*_a_ (21 kJ mol^−1^) of the HER in NH_3_BH_3_(aq) over Pt NPs.^22^ The difference in the activation energy, Δ*E*_a_, between WC–Co_carbide_ and Pt NPs^22^ was 33 kJ mol^−1^, which corresponds to −0.34 V, consistent with the *η*_H2_ of Co.^29^ This analysis indicated that the hydrogen release sites were the Co nanocrystals in the WC matrix. In general, *η*_H2_ is defined as the complex energies consisting of the elementary processes: (I) alignment of H^+^(aq) on the electrode; (II) accepting electrons to form the radical hydrogen atom H(rad.); (III) bonding two H(rad.) to form a H_2_ molecule; (IV) convergence of the radicals to form hydrogen gas H_2_(g); and (V) desorption of H_2_(g) from the electrode. In this study, the separation of these elementary processes of *η*_H2_ was impossible as *E*_a_ was determined only from a simple Arrhenius plot. Further computational simulations are necessary to clarify this point. The *A* value is discussed later with respect to the thermodynamic cycle.

### Magnetic behaviour

**Fig. 5:**
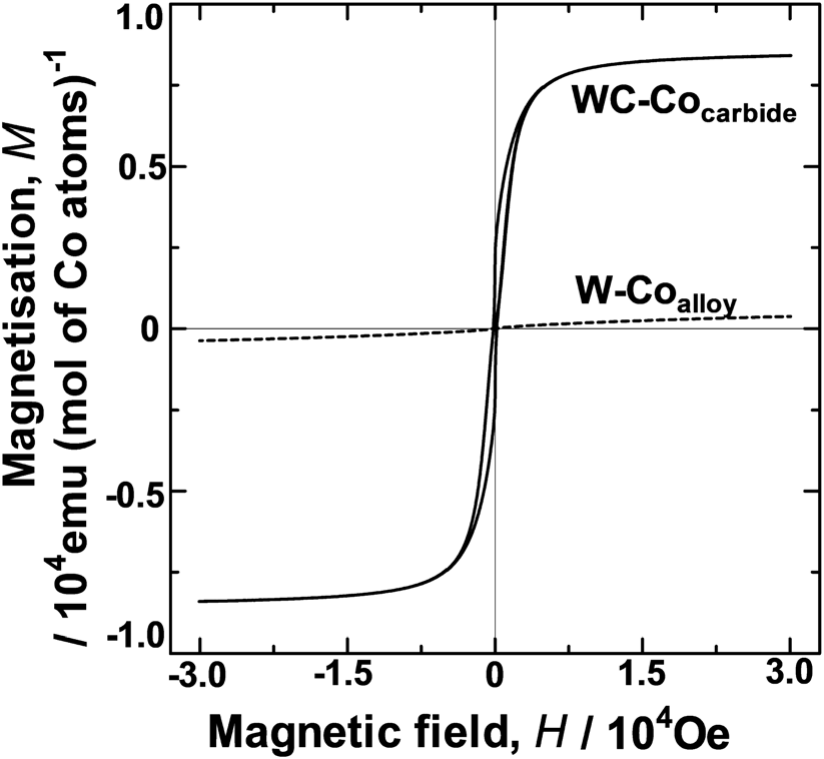
Magnetisation as a function of magnetic field for WC–Co_carbide_ and W–Co_alloy_ at 4 K.

The HER over the W–Co_alloy_ powder was not enhanced as observed for WC–Co_carbide_ (Fig. 2), indicating that H_2_(g) is not readily evolved over the Co atoms substituted into the bcc W lattice. However, the HER was enhanced in the case of the WC–Co_carbide_ sample with Co atoms configured in the nanocrystal domain. The most likely difference between these configurations is related to their magnetic properties. Hence, *M* was investigated to clarify the different HER results. Fig. 5 shows *M* as a function of the magnetic field (*H*) for WC–Co_carbide_ and W–Co_alloy_ at 4 K. WC–Co_carbide_ showed a clear hysteresis loop, indicating that it is a ferromagnetic material. Assuming that the value of *M* for WC–Co_carbide_ resulted from the Co crystals, the maximum saturated value, *M*_S_, was 8.41 × 10^3^ emu (mol Co atoms)^−1^. However, the value of *M* for W–Co_alloy_ was close to zero, indicating that it had a spin glass state.^30^ Similarly, the *M* (H) data were measured at 308 K, yielding similar results to those at 4 K for both samples.

The magnetic moment *β* of a Co atom in the Co nanocrystals in WC–Co_carbide_ at 4 K and 308 K were 1.50 and 1.42 *μ*_B_ per Co atom, respectively, where *μ*_B_ is the unit of the Bohr magneton. In the elemental Co crystal, the value of *β* for a Co atom is 1.7 *μ*_B_.^27^ Hence, the Co nanocrystals had a *β* value close to that of elemental Co. In contrast, the *β* value of a Co atom substituted into the bcc W lattice in the W–Co_alloy_ measured at 4 K and 308 K was 6.79 × 10^−2^ and 7.48 × 10^−3^*μ*_B_, respectively. As described in our previous study,^24^ the XRD pattern of W–Coalloy showed only the peaks of W.^24^ The composition of W–Coalloy corresponds to the two phase equilibria region of the W phase and the intermediate phase of Co_7_W_6_ in the phase diagram of the W–Co binary system.^31^ Co_7_W_6_ has complicated long-range periodic structure.^32^ Formation of the complicated long-range periodic structures such as the sigma phase in the heat-resistant alloys takes a lot of time to accomplish their atomic configuration.^33^ Hence, the equilibrium phase of Co_7_W_6_ was not formed during reduction with H_2_ gas. The Co atoms are concluded to be supersaturated in the W lattice as a nonequilibrium state. Consequently, the spin configuration among isolated Co atoms was random (*i.e.*, a spin glass state^30^). Hence, the spin-ordered state of the Co in WC–Co_carbide_ appears to be one of the factors determining the singular catalytic activity.

Cerri *et al.*^17^ found that hydrogen chemisorption induced a disordering of electronic spin polarisation on the surface of ferromagnetic Gd, resulting in a change in *M*, and the Curie temperature, *T*_C_. It is likely that hydrogen chemisorption relaxes the spin configuration in the system. In aqueous solutions, the spin configuration of the protons is relevant. Here, the protons are likely to be absorbed on the ferromagnetic Co nanocrystals, such that their nucleus spin configurations are aligned to be antiparallel to relax the spin polarisation of the surface.

### Thermodynamic cycle

The following equations show the thermodynamic equilibria among the species associated with the HER from hydrolysis of NH_3_BH_3_(cr), where the standard enthalpies of formation, Δ_f_*H*^°^_m_, at 298.15 K of the standard substances of NH_3_BH_3_(cr),^34^ H_2_O(l),^35^ ammonia (NH_3_(aq)),^35^ orthoboric acid (B(OH)_3_(aq)),^35^ ammonium (NH^4+^(aq)),^35^ metaboric acid (BO_2_^−^(aq)),^36^ and H_2_(g)^35^ are summarised in Table S1.†Eqn (2) shows the hydration reaction of NH_3_BH_3_(cr), where the thermodynamic value is unknown. Eqn (3) shows the HER of the hydrolysis of NH_3_BH_3_(aq). Eqn (4), rewritten as the sum of eqn (2) and (3), indicates the HER from the initial substance of NH_3_BH_3_(cr). Eqn (5) shows the formation of NH^4+^(aq). Eqn (6) shows the formation of BO_2_^−^(aq). Finally, eqn (7), rewritten as the sum of eqn (4)–(6), shows the final state of the hydrolysis.

2 NH_3_BH_3_(cr) = NH_3_BH_3_(aq)

3 NH_3_BH_3_(aq) + 3H_2_O(l) = NH_3_(aq) + B(OH)_3_(aq) + 3H_2_(g)

4 NH_3_BH_3_(cr) + 3H_2_O(l) = NH_3_(aq) + B(OH)_3_(aq) + 3H_2_(g)

Δ_r_*H*^°^/kJ = 3 × Δ_f_*H*^°^_m_(H_2_(g)) + Δ_f_*H*^°^_m_(NH_3_(aq)) + Δ_f_*H*^°^_m_(B(OH)_3_(aq)) − Δ_f_*H*^°^_m_(NH_3_BH_3_(cr)) − 3 × Δ_f_*H*^°^_m_(H_2_(g)) = −118.494 ± 5.921

5 NH_3_(aq) + H^+^(aq) = NH^4+^(aq), Δ_r_*H*^°^/kJ = −52.090 ± 0.411

6 B(OH)_3_(aq) = BO_2_^−^(aq) + H_2_O(l) + H^+^(aq), Δ_r_*H*^°^/kJ = 14.600 ± 0.980

7 NH_3_BH_3_(cr) + 2H_2_O(l) = BO_2_^−^(aq) + NH^4+^(aq) + 3H_2_(g), Δ_r_*H*^°^/kJ = −155.983 ± 6.016

Since eqn (5)–(7) are spontaneous reactions, the HER is given by eqn (4). As the standard entropy, *S*^°^_m_, of NH_3_BH_3_(cr) has not yet been measured, the standard entropy of reaction, Δ_r_*S*^°^, and the standard Gibbs energy of reaction, Δ_r_*G*^°^, are unknown. However, Δ_r_*G*^°^ is more negative than Δ_r_*H*^°^ as the HER increases Δ_r_*S*^°^. Therefore, when a driving energy is applied corresponding to the hydrogen overpotential of Co, the HER reaches equilibrium, as defined by eqn (7)*via*eqn (4).

In previous studies, Co NPs (10 wt% Co on an Al_2_O_3_ support)^21^ had an HER rate 10 times lower than that of Pt NPs (2 wt% Pt on an Al_2_O_3_ support).^22^ However, in the present study, the HER rate of WC–Co_carbide_ was 30% higher than that of Pt_NPs, even if *E*_a_ corresponds to the *η*_H2_ of Co. The excellent catalytic activity was a result of the high *A* value, as determined from the Arrhenius plot (Fig. 4). The WC matrix seems to facilitate the release of H^+^(aq) from NH_3_BH_3_(aq) and contribute to increasing *A* (*i.e.*, the number of H^+^(aq) collisions). In accordance with the electron theory, the Pt-like high DOS near the *E*_F_ of the surface of WC^7^ can induce adsorption of NH_3_BH_3_ molecules, which can promote the decomposition of B–N bonds to form NH_3_(aq), B(OH)_3_(aq), and H_2_(g) *via* highly unstable BH_3_(aq) close to the equilibrium states (see eqn (3)). Furthermore, considering the thermodynamic hierarchy, as shown in Table S1,† the Δ_f_*G*^°^_m_ of WB^37^ is smaller than that of WC,^38^ indicating that the chemical bonding between the W and B atoms is more stable than that between the W and C atoms. In addition, it is well known that natural tungsten ore consists of ammonium tungstate,^39^ which is the starting material used in the present study, indicating the high affinity between tungsten and ammonia. Therefore, W in the WC matrix has a driving force for attracting NH_3_BH_3_(aq), resulting in preferential adsorption. Although a previous study^20^ investigated the hydrolysis kinetics of NH_3_BH_3_ using first principles calculations based on the transition state theory and estimated the atomic distance of a B–N bond, the interaction between the WC matrix and NH_3_BH_3_(aq) should be further investigated by first principles and molecular dynamics calculations. The BH_3_(aq) molecule can release three protons while bonding with three OH^−^ ions to form B(OH)_3_. As shown in Table 1, when the amount of NH_3_BH_3_(aq) was doubled, *N*_HER_ also doubled, indicating that there were sufficient HER reaction sites. Hence, the WC matrix played a crucial role in adsorbing NH_3_BH_3_ molecules and decomposing B–N bonds, followed by supplying protons to the Co crystals.

### Specific enthalpy of hydrogen adsorption

The Δ_ad_*H*^°^_m_ values of WC–Co_carbide,_ and commercial WC and Pt powders at 423 K were −22.42 ± 0.90, −21.87 ± 0.87, and −109.72 ± 4.39 J m^−2^, respectively. We found that the Δ_ad_*H*^°^_m_ of WC was less exothermic than that of Pt, implying that the desorption of H_2_(g) from the surface of WC was not difficult. Previous studies have investigated the catalytic activity of WC^13^ and related carbide composites^11,12^ under an external voltage. When an external voltage is applied, the H^+^(aq) ions align on the surface of WC, followed by hydrogen evolution. However, when no external voltage is applied, such an alignment of H^+^(aq) is unlikely to be significant. In contrast, on the surface of WC–Co_carbide_, the H^+^(aq) ions seem to align as a result of the magnetic field of the Co nanocrystals, followed by hydrogen evolution. The Δ_ad_*H*^°^_m_ of Pt is extremely high, indicating that hydrogen was stored in its lattice. Pt simultaneously achieves the adsorption, storage, and desorption of hydrogen species, although the specific mechanism is still unknown.

### Mechanism of a singular catalytic behaviour of WC–Co_carbide_

**Fig. 6:**
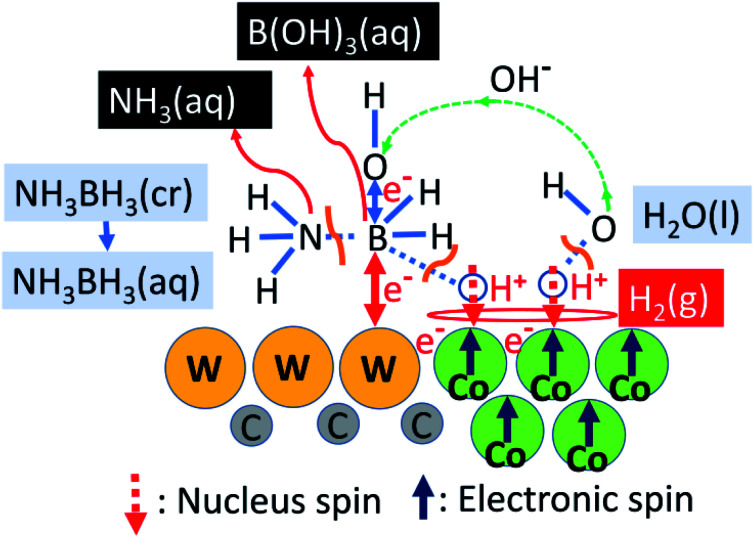
Schematic of hydrogen evolution from the hydrolysis of NH_3_BH_3_(aq) over the WC–Co_carbide_ catalyst.

Fig. 6 shows a schematic of the catalytic HER over WC–Co_carbide_, which involves the following steps. (1) The NH_3_BH_3_ molecules are absorbed on the WC matrix by the attractive interaction of W–B bonds; (2) the B–N bonds in NH_3_BH_3_(aq) are broken to form stable NH_3_(aq) and unstable BH_3_(aq); (3) the B atoms in BH_3_(aq) with a significantly short lifetime release three quasi-stable protons and coordinate three OH^−^(aq) ions from the surrounding H_2_O molecules to form stable B(OH)_3_ molecules, wherein the H_2_O releases three protons; (4) sufficient amounts of protons from BH_3_(aq) and H_2_O are adsorbed on the ferromagnetic Co nanocrystals in the WC matrix, resulting in antiparallel alignment of the nucleus spin configurations of the two H^+^(aq) to relax the spin polarisation of the system; (5) hydrogen molecules are evolved as the protons accept electrons, e^−^, such that *E*_a_ is consistent with *η*_H2_ of Co, wherein Co is protected by galvanic protection;^40^ (6) one e^−^ is released during the decomposition of BH_3_, along with a proton, while the other e^−^ is released from OH^−^(aq) during coordination to form B(OH)_3_(aq), *i.e.* the origin of charge transfer is the B atom adsorbed on the W atom, wherein the charge is transferred to Co *via* the WC matrix; (7) the WC matrix decomposing NH_3_BH_3_(aq) and generating H^+^(aq) applies a driving energy corresponding to the necessary hydrogen overpotential, wherein the WC matrix supplies excess protons to the Co sites.

The relative volume ratio of the WC matrix *vs.* Co nanocrystals is estimated to be 100 : 13 from the densities of pristine WC and Co. When the relative SSA ratio of the WC matrix *vs.* Co nanocrystals is hypothetically equal to the relative volume ratio, the SSA of the Co nanocrystals is 0.15 m^2^ g_cat_^−1^. Assuming the Co nanocrystals only contribute HER, *N*_HER_ is evaluated as 33.84 (H_2_ mol min^−1^ m^−2^) which is twelve times faster than that of Pt_NPs (=2.90 (H_2_ mol min^−1^ m^−2^)). Such an extremely fast *N*_HER_ is never caused by the single Co nanocrystals. Hence, the Pt-like catalytic activity is attributed to the synergistic effect of the WC matrix and ferromagnetic Co nanocrystals.

Previous studies have determined the *E*_a_ values for the HER in NH_3_BH_3_(aq) of 21 kJ mol^−1^ over Pt NPs^22^ and 62 kJ mol^−1^ over Co NPs,^21^ and suggested that the rate determining step (RDS) was the cleavage of the B–N bonds, as unstable BH_3_ reacts with H_2_O to form H_2_. In addition, Wang *et al.*^23^ determined an *E*_a_ value of 42.7 kJ mol^−1^ for the HER in NH_3_BH_3_(aq) catalysed by Ni NPs, and suggested that the RDS was the cleavage of the O–H bond in H_2_O (*E*_a_ = 493 kJ mol^−1^), as the equilibrium bonding energy was more endothermic than that of the B–N (*E*_a_ = 117 kJ mol^−1^) and B–H (430 kJ mol^−1^) bonds. The Δ*E*_a_ between Co NPs^21^ and Pt NPs^22^ and that between Ni NPs^23^ and Pt NPs^22^ were 41 and 21.7 kJ mol^−1^, corresponding to −0.43 and −0.22 V, respectively, thereby demonstrating consistency with the *η*_H2_ of Co and Ni.^29^ These *E*_a_ results support the mechanism depicted in Fig. 6.

Another definition of *η*_H2_ in electrochemistry is the HER current density, *i*_0_, determined by extrapolating the cathodic Tafel line to the reference hydrogen electrode.^41^ Fig. S4† shows the correlation between magnetic susceptibility, *X*,^42^ and log *i*_0_ ^41^ of the transition metals of the fourth, fifth, and sixth periods (rows) of the periodic table of the elements. In the sixth period, the log *i*_0_ value of Pt was large, consistent with the high HER catalytic activity, while *X* was large, corresponding to a high log *i*_0_. Similarly, in the fifth period, the log *i*_0_ values and *X* values of Rh and Pd were large. In the fourth period, the log *i*_0_ values of Fe, Co, and Ni were high. As these metals are ferromagnetic with spontaneous magnetisation, their *X* values are not relevant. However, their spin-ordered ferromagnetic state likely increased their log *i*_0_ values. In contrast, the diamagnetic transition metals of Au and Hg in the sixth period, Ag and Cd in the fifth period, and Cu and Zn in the fourth period with negative *X* values showed small log *i*_0_ values consistent with their lower HER catalytic activities. Therefore, we conclude that the magnetic properties of the transition metals are related to the HER catalytic activity. Materials with high *X* values are likely to interact readily with the nucleus spin of H^+^(aq). Ferromagnetism seems to induce alignment of the nucleus spin of H^+^(aq) on the ferromagnetic metal surface, as discussed with regard to the contribution of the Co nanocrystals in this study. After formation of two radical H atoms by donation of electrons, the spin conversion of the two H atoms should form the 1*σ*_g_ molecular orbital of a H_2_ molecule. The magnetic properties of metals are likely to contribute to the spin conversion. After H_2_ molecules are formed, they are released from the surface of the metals with a high positive *X* value or ferromagnetism because H_2_ is diamagnetic with a negative *X* value (−19.7 × 10^−9^ m^3^ kg^−1^).

Recently, it was shown that an external magnetic field enhanced the oxygen evolution reaction (OER) during electrolysis of a KOH aqueous solution.^19^ A Pt plate was used as the cathode and ferrite NiZnFe_4_O_*x*_ (deposited on Ni foam by direct magnetic interaction between the materials) was used as the anode. An external magnetic field of 0.45 T was applied to the anode using a rare-earth permanent magnet, which resulted in the OER current almost doubling (from 24 to 40 mA cm^−2^ at ≥1.65 V).^19^ They hypothesised that the external voltage accelerated the spin conversion to form the paramagnetic triplet of O_2_(g). Li *et al.*^43,44^ prepared a series of metal–organic frameworks (MOFs) composed of the Fe–Ni binary^43^ and W–Co–Fe ternary system^44^ as electrocatalysts. Their superior catalytic properties appear to result from the magnetic elements of Fe,^43,44^ Ni^43^, and Co.^43,44^ These findings are highly relevant to the present study.

There have been many investigations of the electronic states of HER.^29^ However, the interaction between the nucleus spin of H^+^(aq) and electronic spin of metals has not yet been investigated. Thomas *et al.*^45,46^ calculated the energy eigenvalues of the electrons and nuclei of hydrogen atoms (protons) by considering the wave functions of both protons and electrons, and describing the zero-point motion of the protons by a Slater-type wave function. This approach could be applied to the HER catalytic reaction.

As described above with respect to the thermodynamics, the WC matrix appears to facilitate the release of H^+^(aq) from NH_3_BH_3_(aq) and contribute to increasing *A* (*i.e.*, the number of collisions between H^+^(aq) ions). However, H_2_(g) appears to be barely formed by such collisions. The alignment of H^+^(aq) on the catalyst surface and the donation of an electron to the ion are necessary. When an external electric voltage is applied to WC, these requirements are likely to be satisfied, resulting in the catalytic activity observed in previous studies.^11–16^ The internal magnetic field is considered sufficient to induce catalytic activity without requiring an external voltage.

Pt seems to easily adsorb NH_3_BH_3_(aq) with the highest DOS at the *E*_F_ of all elements, and easily evolves H_2_(g) from its surface with the smallest *η*_H_2__ of any known element. A study of the interaction between Pt with a high magnetic susceptibility and protons with a nucleus spin appears to be a necessary future work to consider the effect of the magnetic field from the earth on the spin alignment of the protons.

Pt catalysts have low reusability in HER from HCOOH due to CO poisoning. WC–Co_carbide_ is advantageous for avoiding CO poisoning due to that it is prepared by carburizing with CO *via* CO_2_. Reusability of WC–Co_carbide_ should be further investigated.

## Conclusion

The catalytic activities of WC and its composites had previously only been observed under the conditions of an applied external voltage, and the predicted intrinsic catalytic activity of mono WC without an external voltage had not been verified. We hypothesised that the introduction of an internal magnetic field would provide conditions similar to those provided by the external voltage to enhance the catalytic performance of WC. To this end, the WC lattice was doped with ferromagnetic Co nanocrystals to introduce an ordered-spin configuration as an internal magnetic field. The internal magnetic field successfully increased the HER rate during hydrolysis of NH_3_BH_3_ to a value even higher than that of the Pt nanoparticles. The enhanced catalytic activity was attributed to the synergistic effect of the WC matrix promoting hydrolytic cleavage of NH_3_BH_3_ and the ferromagnetic Co crystals interacting with the nucleus spin of the protons. Our new strategy for enhancing the catalytic performance by introducing an internal magnetic field is expected to provide new opportunities for developing novel catalysts, such as those for hydrolysis of NH_3_BH_3_ and the electrodes of fuel cells.

## Conflicts of interest

There are no conflicts to declare.

## Supplementary Material

RA-011-D1RA01181B-s001
