# Supplementary material for: Catalytic activity of Co-nanocrystal-doped tungsten carbide arising from an internal magnetic field
Source: RSC Adv. 2021 Apr 14;11(23):14063–70. doi: 10.1039/d1ra01181b (PMC8697676; doi:10.1039/d1ra01181b)
Supplement: RA-011-D1RA01181B-s001 [file RA-011-D1RA01181B-s001.pdf]

Electronic Supplementary Material (ESI) for RSC Advances.
This journal is © The Royal Society of Chemistry 2021

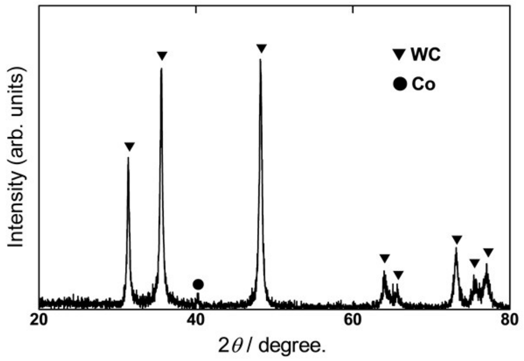

Fig. S1 XRD pattern of WC-$Co_{carbide}$ prepared by carburization of W-Co_{alloy} powder at 1173 K for 32.4 ks.

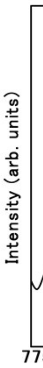

Fig. S

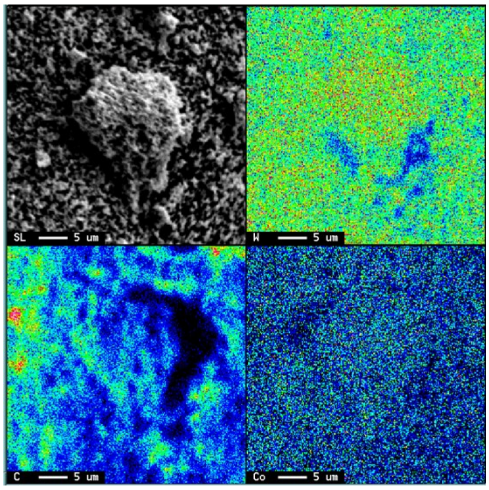

Fig. S3 EPMA images of WC-$Co_{carbide}$ prepared by carburization of W-Co_{alloy} powder at 1173 K: SEM image; W $L\alpha$ image; C $K\alpha$ image; Co $K\alpha$ image.

Table S1 Summary of the thermodynamic values at 298.15 K and their uncertainties, *U*, of the substances of interest.

Substances	$\Delta_f H_m^\circ$/(kJ (mol of compd.)$^{-1}$)	$U(\Delta_f H_m^\circ)$/(kJ (mol of compd.)$^{-1}$)	S_m°/(J K^{-1} (mol of compd.)$^{-1}$)	$U(S_m^\circ)$/(J K^{-1} (mol of compd.)-^{1})	$\Delta_f G_m^\circ$/(kJ (mol of compd.)$^{-1}$)	$U(\Delta_f G_m^\circ)$/(kJ (mol of compd.)$^{-1}$)	Refs.
WC(cr)	-40.166	-	32.384	-	-38.372	-	(38)
WB(cr)	unknown	-	unknown	-	-123.82	± 4.95[a]	(37)
NH_3BH_3(cr)	-178.0	± 5.9	unknown	-	unknown	-	(34)
NH_3(aq)	-81.170	± 0.326	109.040	± 0.913	-26.673	± 0.305	(35)
NH^{4+}(aq)	-133.260	± 0.250	111.170	± 0.400	-79.398	± 0.278	(35)
$B(OH)_3$(aq)	-1072.800	± 0.800	162.400	± 0.600	-969.268	± 0.820	(35)
BO_2^-(aq)	-772.37	± 0.56[b]	-37.2	± 0.25[b]	-678.89	± 0.57[b]	(36)
H_2O(l)	-285.830	± 0.040	69.950	± 0.030	-237.140	± 0.041	(35)
H_2(g)	0.000	± 0.000	130.680	± 0.003	0.000	± 0.000	(35)

[a] Extrapolated data of the temperature function $\Delta_f G_m^\circ$(WB) at 1310 – 1399 K,[37] giving $U(\Delta_f G_m^\circ)$ of 4%.

[b] Uncertainties of BO_2^-(aq) were estimated to be equal to those of $B(OH)_3$(aq).

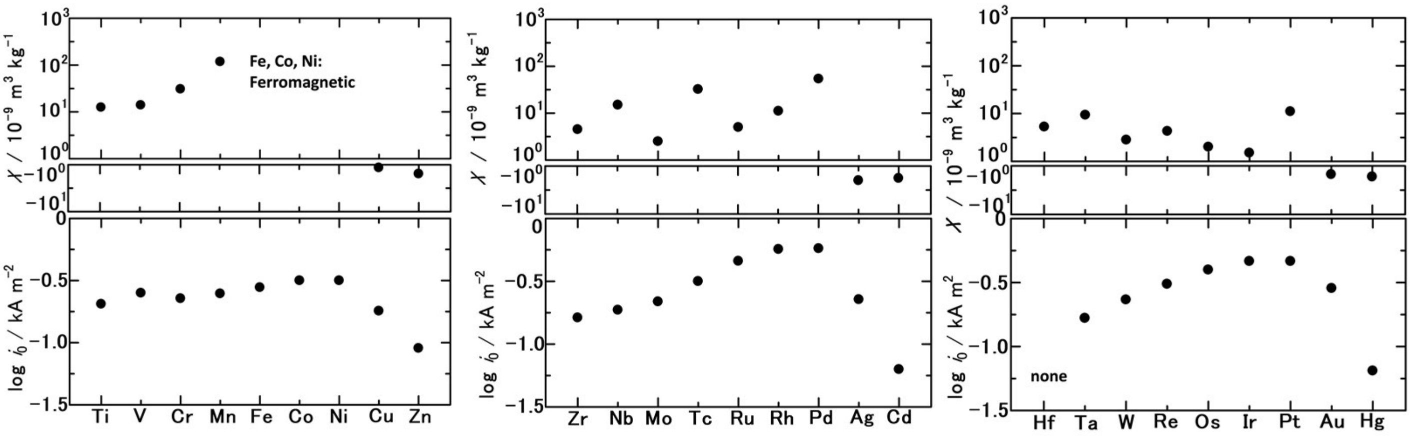

Fig. S4 Correlation between magnetic susceptibility, *X*, and electrochemical HER current density i_0 determined by extrapolating the cathodic Tafel line to the reference hydrogen electrode (RHE) of the transition metals of the fourth, fifth and sixth periods.
